# Network Pharmacology, Molecular Docking, and Molecular Dynamic-Based Investigation on the Mechanism of Compound Chrysanthemum in the Treatment of Asthenopia

**DOI:** 10.1155/2022/3444277

**Published:** 2022-12-30

**Authors:** Junjie Qiu, Biying Zheng, Hengpu Zhou, Chengcong Ye, Menglin Shi, Senlin Shi, Suxiang Wu

**Affiliations:** School of Pharmaceutical Sciences, Zhejiang Chinese Medical University, Hangzhou 311402, China

## Abstract

As a clinical empirical prescription for ophthalmology, compound chrysanthemum has been used gradually and has a good effect on eye fatigue. However, the detailed mechanisms of antiasthenopia have not been studied. In order to clarify the mechanisms of the compound chrysanthemum in the treatment of asthenopia, network pharmacology was combined with experimental study in this paper. A total of 593 genes and 39 active chemicals were identified, and both were considered to be essential to the advancement of asthenopia research. The results of the molecular docking analysis demonstrated a certain affinity between PRKACA, PRKCA, PRKCB, and their related compounds; molecular dynamic simulations assessed the stability of these receptors and ligands. The effects of compound chrysanthemum extract on ciliary muscle were studied in vitro and in vivo. By using the MTT assay, compound chrysanthemum extracts (50, 100, 200, 400, and 800 g·mL-1) showed no effect on the proliferation of rCSMCs for 24 and 48 hours. It raised nitric oxide and decreased Ca^2+^ in ciliary muscle cells isolated from the eyeballs of rats. Besides, compound chrysanthemum extract had a direct relaxing effect on the isolated gastric smooth muscle of rats by reducing the contractile tension. Furthermore, in vivo experiment results showed that, compared to the incandescent lamp-irradiated rats (model group), SD rats treated with compound chrysanthemum extracts (660 mg·kg^−1^ and 1320 mg·kg^−1^, orally) displayed considerably retracted pupils and increased NO content. It is also found that compound chrysanthemum extract can downregulate the mRNA expression of PKA and PKC in the calcium signaling pathway. Overall, our results suggested that compound chrysanthemum extract may lessen visual fatigue through multiple components, multiple targets, and multiple pathways.

## 1. Introduction

Asthenopia is one of the most common eye diseases on the ophthalmology. After utilizing the eyes for a prolonged period of time, it may result in symptoms like vision disruption and eye discomfort. In severe cases, it can even deteriorate into systemic symptoms [1–4]. Clinically speaking, visual fatigue is categorized as a syndrome because it might develop due to local, systemic, mental, or psychological reasons [5, 6]. The main manifestations of symptom include redness, soreness, itching, overflow of tears, diplopia, headache, dry eyes, foreign body sensation, and nausea [7–9].

The compound chrysanthemum (CC) is composed of *Chrysanthemi Flos*, *Cassiae Semen*, *Lycii Fructus*, *Polygonati Rhizoma*, *Ligustri Lucidi Fructus*, and *Ecliptae Herba*. It is a clinically proven prescription that has been used extensively in ophthalmology and has good curative benefits. Chrysanthemum and *Lycii Fructus*, as traditional Chinese herbs, always have the effect of brightening the eyes and relieving asthenopia [10]. Studies have proved that chrysanthemum has a strong antioxidant effect and can slow down retinal damage [11]. In Japan, these 2 herbs are also widely used to treat eye fatigue [12]. The 2-O-*β*-D-glucosyl-L-ascorbic acid in *Lycii Fructus* can improve the antioxidant capacity of oxidatively damaged lens and slow down the damage of lens to maintain the transparency of lens [13, 14]. The active ingredients such as flavonoids, terpenoids, and organic acids in chrysanthemum have pharmacological effects on relieving fatigue, protecting liver, regulating immunity, etc. [15–19]. Chrysanthemum also can inhibit the apoptosis of lacrimal gland acinar cells, lacrimal gland duct cells and improve the basic secretion of tear fluid to maintain the stability of the tear film. It may be one of the mechanisms of chrysanthemum improving the symptoms of dry eyes [20]. *Cassiae semen* contains a variety of active ingredients such as anthraquinones and polysaccharides, which have the effects of protecting the liver, scavenging free radicals, and enhancing immunity [21, 22]. The polysaccharides, flavonoids, and other active components of *Lycii Fructus* provide pharmacological effects such as liver and kidney protection and tiredness relief [23–26]. The pathological changes of glaucoma and other eye illnesses are also well-adjusted by it [27]. The main components of *Polygonati Rhizoma* are polysaccharides, anthraquinones, alkaloids and steroidal saponins, etc., which have the effects of regulating immunity, antitumor, antifatigue, and inhibiting bacteria. *Ligustri Lucidi Fructus* is rich in triterpenoids and flavonoid, which hold potential to antiasthenopia. There have been multiple studies which indicate that Wedelolactone in *Ecliptae Herba* plays a role in inhibiting hepatocyte apoptosis and liver damage [28–31].

In recent years, computer technology has been widely used to solve medical problems [32, 33]; after we find possible targets for some diseases, like dengue, tuberculosis [34, 35], etc., we can use molecular docking technology to predict the binding energy of active compounds and potential targets; molecular dynamics simulation technology can simulate the effect of active compounds and potential targets after binding. These computer technologies can help us more effectively explore the mechanism of drug treatment to diseases [36, 37], but experimental verification is essential.

**Figure 1:**
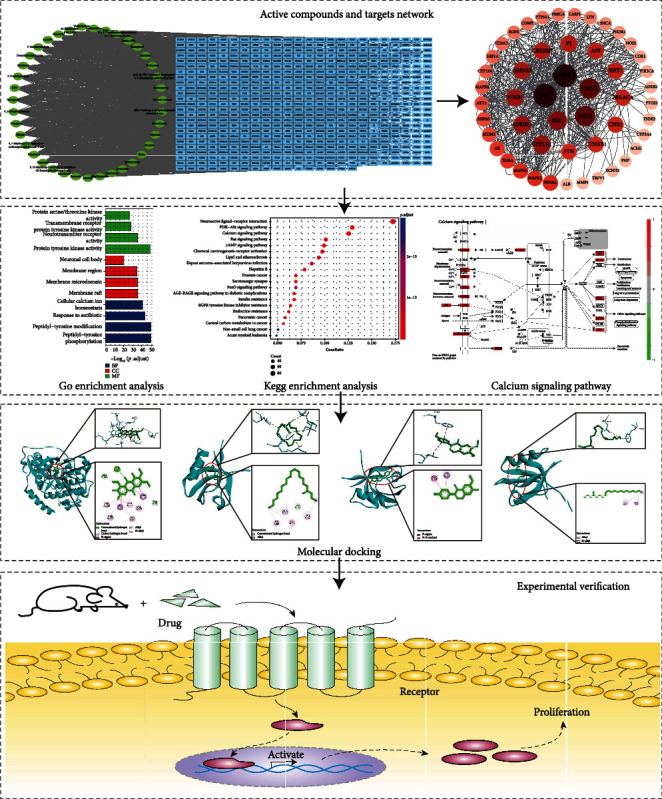
Illustration of CC in treatment of asthenopia.

In this study, we used network pharmacology to explore the mechanism and signaling pathways of CC against asthenopia. Subsequently, the binding affinity and the stability of the targets to the active CC compounds were predicted using molecular docking and molecular dynamic. Additionally, the results of the cell and animal studies demonstrated that CC can increase the production of NO, decrease the content of Ca^2+^, and reduce the mRNA expression of PKA and PKC, so as to achieve the therapeutic effect of relaxing the ciliary muscle. The flowchart of the whole study design is illustrated in Figure 1.

## 2. Materials and Methods

### 2.1. Chemicals and Reagents

QijuDihuang oral liquid (QJDHOL) was purchased from Hubei Dongxin Pharmaceutical Co., Ltd. Other Medicinal herbs were purchased from Zhejiang Chinese Medical University Chinese Medicine Decoction Pieces Co., Ltd. rCSMCs were purchased from Shanghai Qincheng Biotechnology Co., Ltd. Australian Fetal Bovine Serum and RPMI 1640 was purchased from Thermo Fisher Scientific-CN. Griess reagent was purchased from Shanghai Yuanye Bio-Technology Co., Ltd. Pluronic F127 and Fura-2/AM (5 *u*m) was purchase from Shanghai Yisheng Biological Technology Co., Ltd. MonScript™ RTIII Super Mix with dsDNasekit and MonAmp™SYBR® Green qPCR Mix kit for qRT-PCR were bought from Monad Biotechnology Co., Ltd. (Wuhan, China). RNAeasy™Plus Animal kit was purchased from the Beyotime Institute of Biotechnology (Jiangsu, China). Other chemicals were obtained from Tianjin Yongda Chemical Reagent Co., Ltd.

### 2.2. Network Pharmacology-Based Analysis

#### 2.2.1. Screening the Effective Components and Potential Targets of CC

The Traditional Chinese Medicine Systems Pharmacology Database and Analysis Platform (TCMSP, https://old.tcmsp-e.com/tcmsp.php) was utilized to screen active ingredients of CC by using “Juhua,” “Juemingzi,” “Gouqizi,” “Huangjing,” “Nvzhenzi,” and “Mohanlian” as keywords, with the criteria OB ≥ 30% and DL ≥ 0.18 [38]. With good gastrointestinal absorption ((GI) absorption) capabilities in the pharmacokinetics column and at least two yeses for the first five conditions in the druglikeness column, the acquired components were analyzed using the SwissADME database [39]. Subsequently, the Swiss Target Prediction platform generated the relevant targets based on the active substances screened.

#### 2.2.2. Prediction of Potential Targets of Asthenopia

Using “asthenopia,” “eye fatigue,” and “visual fatigue,” as the keywords, asthenopia-related targets were gathered from the GeneCards database (https://www.genecards.org/), Online Mendelian Inheritance in Man (OMIM) database (https://www.omim.org/), and Therapeutic Target Database (TTD) (http://db.idrblab.net/ttd). By importing the CC active ingredient targets and asthenopia related targets into VENNY2.1 (https://bioinfogp.cnb.csic.es/tools/venny/), their common targets were obtained [40].

#### 2.2.3. Construction of a Protein-Protein Interaction (PPI) Network and Active Compound-Disease Target Network

With the help of the online software Search Tool for the Retrieval of Interacting Genes/Proteins (STRING) (https://string-db.org/), the PPI network was obtained. The human species was selected, and the confidence value is set to highest confidence in the parameter setting [41]. Subsequently, The PPI network was visualized with Cytoscape 3.7.0 software, and the score of each node was calculated using the CytoNCA, a Cytoscape plugin. Core network were detected according to betweenness centrality (BC) values, and the top 50 genes generated were considered as hub genes; to construct the core network, the credibility of the interaction between the target proteins increases with score. In the meanwhile, to construct an active compound-disease target network, the active compounds from CC and the relevant targets were imported into Cytoscape.

#### 2.2.4. GO and KEGG Pathway Enrichment Analysis

The Gene Ontology (GO) analysis is used to describe the biological functions of targets which include three aspects: biological processes (BP), molecular functions (MF), and cellular components (CC). The Kyoto Encyclopedia of Genes and Genomes (KEGG) is a database which has the ability to perform functional enrichment analysis power. The clusterProfiler software package of the R platform was used to conduct GO and KEGG functional enrichment analysis, with a considering threshold of *p* < 0.05 [42].

#### 2.2.5. Molecular Docking

Using molecular docking analysis, the binding situation of protein and small molecules can be predicted. The significant genes, PRKACA, PRKCA, and PRKCB, were selected to conduct molecular docking. We obtained the crystal structures of PRKACA (PDB ID: 2GU8), PRKCA (PDB ID: 2GZV), and PRKCB (PDB ID: 2I0E) from the Protein Data Bank (PDB) database of the Research Collaboratory for Structural Bioinformatics (RCSB). Firstly, minimizing the target protein free energy with Chem3D, the target proteins were introduced into AutoDock-Tools (Version: 1.5.7), where water molecules and original ligands in the target proteins were removed, and hydrogen atoms were added, then docking box generation within 40 Å with the original ligand as the center. The active ingredients that can bind to these targets were used as ligands, and the 3D molecular conformations of the ingredients were then obtained from the PubChem Compound database [43]. Subsequently, the docking experiments were achieved with the help of AutoDock Vina in a semiflexible way and Discovery Studio software was used to visualize the results.

#### 2.2.6. Molecular Dynamic Simulations (MDs)

After molecular docking, Discovery Studio 2019 was used to verify the reliability of the results. The complexes were first structured automatically using the macromolecule tool, including building loops and protonating. The receptor-ligand complexes were then put into a CHARMm forcefield, and explicit periodic boundary model was chosen; meanwhile, 0.145 M NaCl was added to neutralize the system. Subsequently, running the “standard dynamic cascade” mode, this process consists of five steps: minimization, minimization 2, heating, equilibration, and production. The specific parameters were set as follows: the system temperature rose from 50 k to 300 k, 500 ps for equilibration at target temperature, 10000 ps for production; all of the above time steps were 2 ps; the rest of the parameters remained as default [37, 44].

### 2.3. Experimental Verification

#### 2.3.1. Preparation of Drugs

The parched extract was obtained by the optimal refluxing process and drying process based on the preliminary research of our laboratory, which contained ethanol extract and aqueous extract. The Krebs solution was used to prepare liquid medicines with concentrations of 21.75 mg·mL^−1^, 43.5 mg·mL^−1^, and 87 mg·mL^−1^.

Weigh the medicinal materials according to the prescription of compound chrysanthemum and then decoct them twice with ten times the amount of distilled water. Mix the medicinal solution and dry it into an extract. Prepare the decoction at a concentration of 43.5 mg·mL^−1^ with Krebs' solution. According to the drug instructions, the daily dose of 1.8 mL·kg^−1^ QJDHOL for rats was converted from the dose for adults. Dilute it to a solution with a concentration of 0.075 mg·mL^−1^ by adding a certain volume of Krebs' solution.

#### 2.3.2. Cell Viability

rCSMCs were plated at 5 × 10^4^ mL^−1^ in 96-well plates and treated with compound chrysanthemum extract (CCE) at a concentration of 50, 100, 200, 400, and 800 *μ*g·mL^−1^ for 24 h and 48 h. Then, 20 *μ*L MTT solution at a concentration of 5 mg·mL^−1^ was added to each well. After incubating for 4 h at 37°C, 200 *μ*L DMSO was added to each well of the plates that aspirated the supernatant. The cell viability measured for the absorbance at 490 nm on an enzyme-labeled instrument after shaking for about 15 min. The following formula was used to compute the growth inhibition rate:

(1) $$\begin{array}{c} \text{Growth inhibition rate}=\left[\frac{\left({\text{OD}}_{\text{control}}–{\text{OD}}_{\text{administration}}\right)}{{\text{OD}}_{\text{control}}}\right]\times 100\%. \end{array}$$

#### 2.3.3. Determination of NO Production

The NaNO_2_ solutions with concentrations of 0, 20, 40, 60, 80, 100, 120, 140, 160, 180, and 200 *μ*mol·L^−1^ were prepared to establish a standard curve of NO. The rCSMCs in logarithmic growth phase were treated with CCE at 200, 400, and 800 *μ*g·mL^−1^ for 24 h and 48 h after being starved for 4 h. The level of NO generation was ascertained by detecting nitrite, a stable byproduct of NO interaction with Griess's reagent, in the culture supernatant. Using a microplate reader, the absorbance of the reaction product was measured at 540 nm to determine the nitrite concentration.

The Griess reagent A solution: 0.1% N-1-naphthalene ethylenediamine hydrochloride aqueous solution.

The Griess reagent B solution: 1% sulfaphosphoric acid aqueous solution, *φ*(*H*3PO4) = 5%.

#### 2.3.4. Measurement of Ca^2+^

The rCSMCs in logarithmic growth phase were incubated at 37°C for 60 min with HEPES buffer containing Fura-2/AM (5 *μ*M) and Pluronic F-127 (0.001%) after being rinsed with HEPES buffer 3 times. Then, the supernatant was aspirated, and the rCSMCs were rinsed with HEPES buffer for 3 times, which was followed by CCE treatment (200, 400, and 800 *μ*g·mL^−1^) for 100 s to confirm changes in Ca^2+^ content. Using excitation wavelengths of 340 nm and 380 nm and emission wavelengths of 510 nm, a microplate reader was used to measure the fluorescence intensity of the cells. Fura-2/AM was used as the fluorescent marker of Ca^2+^, and the fluorescence intensity ratio indicated the content of Ca^2+^ in rCSMCs.

#### 2.3.5. Measurement of Muscle Tension

The stomach of the Sprague Dawley (SD) rats was dissected and soaked in Krebs' solution for maintaining its activity. The longitudinal muscles of the stomach were cut out on a glass slide infiltrated with Krebs' solution. The gastric muscle strip specimens about 2.0 cm in length and 0.3 cm in width were cut out with its ends knotted with sutures after removing the mucosal layer. The specimen was placed in a constant temperature water and bathed with 15 mL Krebs' solution and oxygen. One end of it was fixed on a thin rod, and the other end was connected to a tension transducer. The changes in muscle tension were measured by MPA2000 Biosignal Quantitative Recording and Analysis System.

Fresh Krebs solution was replaced into the water bath every 15 minutes to stabilize the specimen for 1 h. The muscle tension (X) before administration was recorded after the specimen was stabilized. After 5 minutes, 6 mL Krebs' solution, QJDHOL, CCE, and decoction were added to different water baths, which ensured that the final concentrations of the positive control group, the CCE low-dose group, the CCE medium-dose group, the CCE high-dose group, and the decoction group in the water bath were 0.0214 mg·mL^−1^, 6.2 mg·mL^−1^, 12.4 mg·mL^−1^, 24.8 mg·mL^−1^, and 12.4 mg·mL^−1^. Changes in muscle tension were observed and recorded after 5 minutes of drug action. The formula for the change rate of muscle tension is as follows:

(2) $$\begin{array}{c} \text{Variation Rate of Muscle Tention}\left(\%\right)=\left[\frac{\left({\text{X}}_{\text{before}}-{\text{X}}_{\text{after}}\right)}{{\text{X}}_{\text{before}}}\right]\times 100\%. \end{array}$$

#### 2.3.6. Antiasthenopia Effect of CCE In Vivo

Male SD rats (300 ± 20 g) provided by Shanghai BK company (the quality certificate number is SYXK (Zhe) 2018-0012) were divided into 6 groups according to weight, which were fed adaptively for 7 days with normal water and food at a temperature of 25°C and relative humidity of 50 ± 20%. The control group was given distilled water for 3 days without glare, and the model group was irradiated with incandescent lamp (100 w) for 15 min without administration. The positive control group received QJDHOL for 3 days at a dose of 1.8 mL·kg^−1^. The CCE was administered orally at 330 mg·kg^−1^, 660 mg·kg^−1^, and 1320 mg·kg^−1^ for 3 days, and then the rats were irradiated with incandescent lamp (100 w) for 15 min. The state of the rat's eyeballs was observed under a slit lamp after the illumination.

The pupil diameter and the eyelid diameter of the rats were measured, and the ratio named pupil diameter/eyelid diameter was calculated. After sacrifice, the eyeballs of rats were immediately enucleated and washed in PBS. The ciliary smooth muscles were carefully separated on ice and weighed. The supernatant was homogenized and centrifuged, and then the Griess assay was used to quantify the amount of NO in the ciliary smooth muscles.

#### 2.3.7. Quantitative Real-Time PCR (qRT-PCR)

Total RNA was extracted from SD rats in accordance with the kit's instructions. Next, RNA samples were reverse transcribed into cDNA and used as a template for PCR amplification. The following conditions for PCR amplification were used: predenaturation at 95°C for 30 sec, 40 cycles of denaturation at 95°C for 10 sec, and annealing at 60°C for 30 sec. The internal reference was GAPDH. The data were analyzed by the 2^−ΔΔCt^ method [45, 46].

#### 2.3.8. Statistical Analysis

The statistical analysis were performed using the SPSS 20.0 software, and the results were expressed as mean ± SD. Differences between groups were analyzed by ANOVA one-way analysis of variance. Statistical significance was set at *p* < 0.05 and *p* < 0.01.

## 3. Results

### 3.1. Identification of Active Compounds and Potential Targets

**Figure 2:**
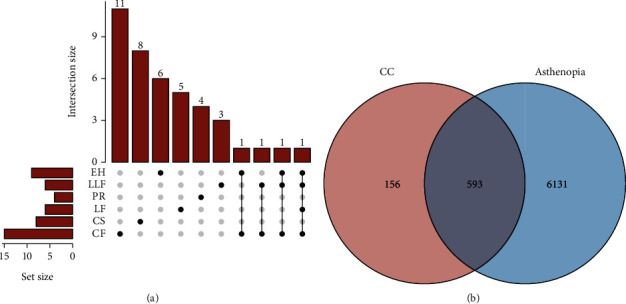
Compounds and targets screening. (a) Compounds in six herbal medicines in compound chrysanthemum. (b) Venn diagram of potential targets of CC for the treatment of asthenopia.

Using the TCMSP database and ADME database, 39 active compounds were obtained in CC (Table 1).  Figure 2(a) showed 11 of CC belonged to *Chrysanthemi Flos*, 8 to *Cassiae Semen*, 5 to Lycii *Fructus*, 4 to *Polygonati Rhizoma*, 3 to *Ligustri Lucidi Fructus*, and 6 to *Ecliptae Herba*. Based on the 39 active compounds, 749 targets were found in the Swiss Target Prediction database. Additionally, 12004 asthenopia targets were gathered from the GeneCards, OMIM, and TTD databases. Then, removing duplicate targets, 6724 potential targets remained. By the online tool Venny 2.1.0 software, an intersection of compound-related targets and disease-associated targets were remained, which contained 593 targets (Figure 2(b)).

### 3.2. PPI Network Analysis

**Figure 3:**
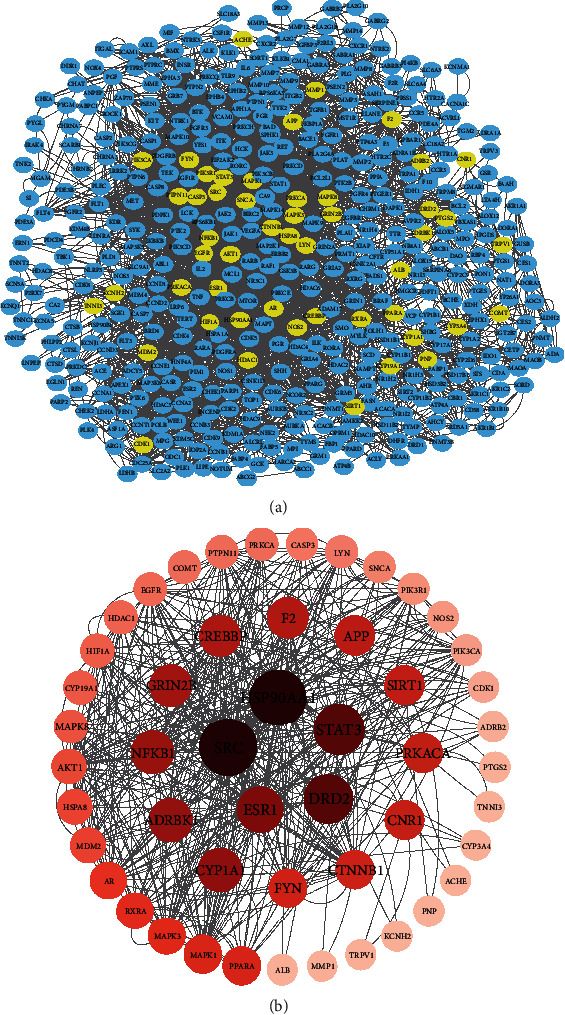
Identification of intersection target network. (a) The PPI network of all common targets. (b) The PPI network of top 50 targets.

To identify central targets, we constructed a PPI network, which may comprehensively elucidate the possible mechanism of CC treatment for asthenopia. There were 593 nodes and 2293 edges in the network (Figure 3(a)). Based on their betweenness centrality values, the top 50 nodes were selected to build a subnetwork (Figure 3(b)); it can be seen that the betweenness centrality values of SRC (298.3), HSP90AA1 (283.6), STAT3 (219.0), DRD2 (212.6), and ESR1 (168.6) were the largest, which were likely to be the hub targets in the progression of asthenopia.

### 3.3. Compound-Disease Target Network

**Figure 4:**
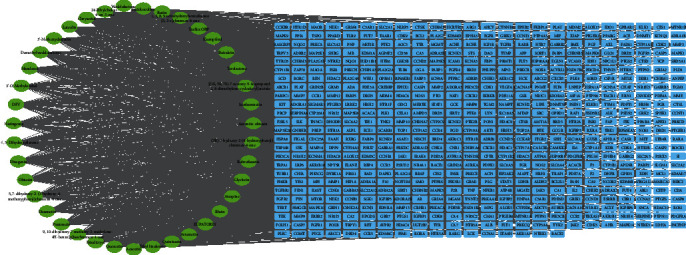
Identification of active compound-potential target network.

The active compound-disease target network was constructed to better understand the probable mechanism of CC on asthenopia. As shown in Figure 4, the network had 632 nodes with 3330 edges. The network showed the potential interaction between compounds and disease targets, which revealed the probable mechanism of the CC in the treatment of asthenopia. With this network, we were able to obtain 39 active compounds related to asthenopia, with an average degree value of 85.38. According to the BC value, the top 5 active compounds were Diosgenin, Lucidusculine, 24-Ethylcholest-4-en-3-one, Rhein, and Truflex OBP.

### 3.4. GO Enrichment Analysis

**Figure 5:**
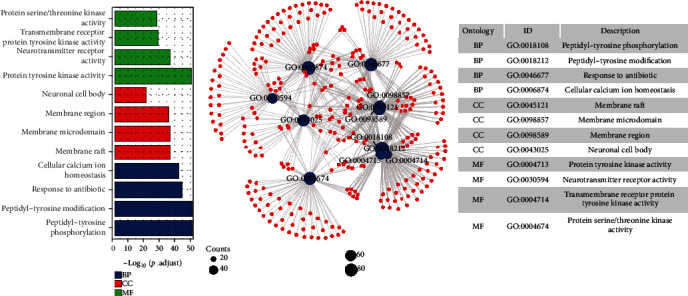
Top 10 significantly enriched terms in BP, CC, and MF of GO analysis.

GO enrichment analysis was used to discover the underlying BPs, CCs, and MFs of the 593 target proteins (Figure 5). The results showed that these proteins were related to 3552 biological processes. Among them, peptidyl-tyrosine phosphorylation, peptidyl-tyrosine modification, and antibiotic response and cellular calcium ion homeostasis ranked the top four. There were 195 cellular components obtained, which membrane raft, membrane microdomain, membrane region, and neuronal cell body were mainly related. In terms of molecular functions, a total of 333 MFs were identified, the top MFs were significantly enriched in protein tyrosine kinase activity, neurotransmitter receptor activity, transmembrane receptor protein tyrosine kinase activity, and protein serine/threonine kinase activity.

### 3.5. KEGG Enrichment Analysis

**Figure 6:**
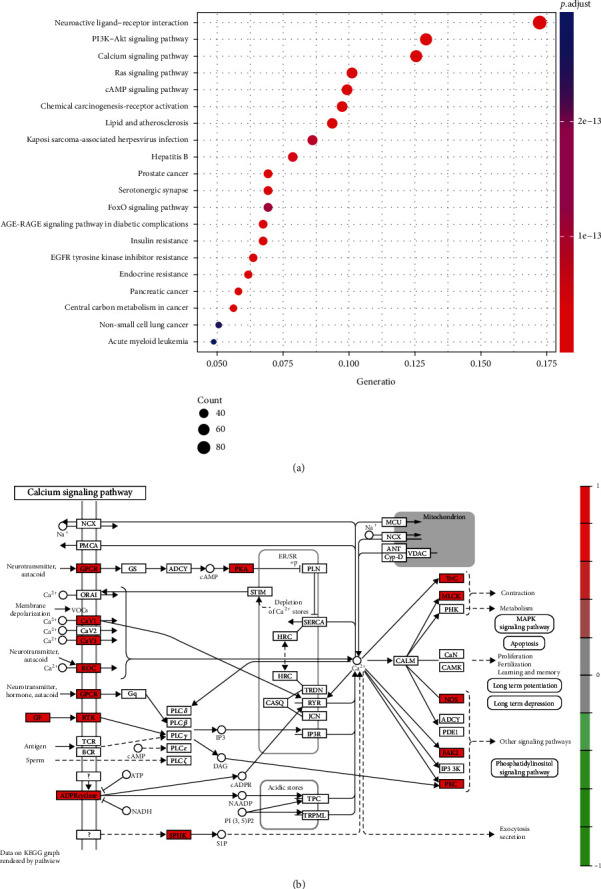
KEGG enrichment analysis and pathway map. (a) The top 20 pathways in KEGG enrichment. (b) Calcium signaling pathway.

KEGG pathway analysis was performed for the 593 target proteins, and a total of 186 KEGG pathways were significantly enriched. The top 20 signaling pathways were chosen for visual display (Figure 6(a)). The hub targets were shown to be considerably enriched in neuroactive ligand-receptor interaction, the PI3K-Akt signaling pathway, and the calcium signaling pathway, suggesting that CC may treat asthenopia by regulating these signaling pathways. The calcium signaling pathway was shown in Figure 6(b).

### 3.6. Validation by Molecular Docking

**Figure 7:**
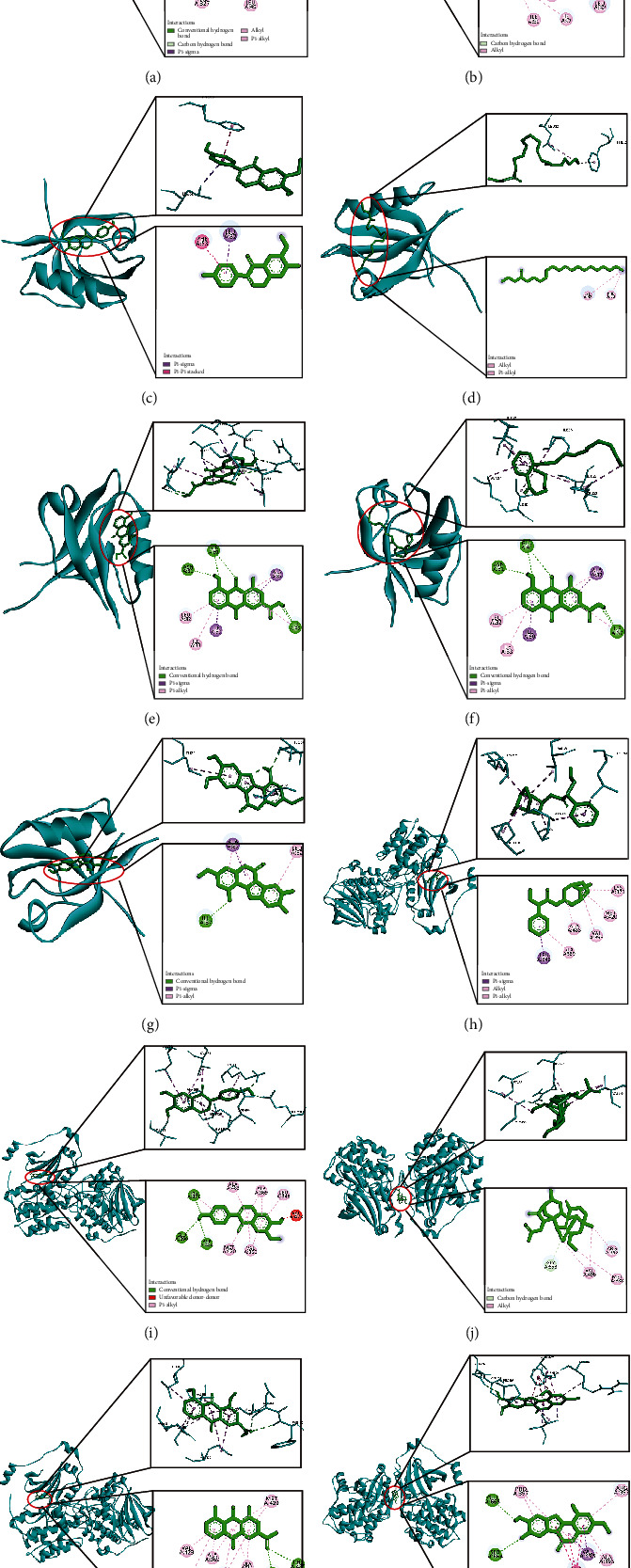
Molecular docking of active compounds and hub targets. (a) The 2D and 3D structure diagrams of PRKACA and Obtusin interaction. (b) The 2D and 3D structure diagrams of PRKCA and Ethyl linolenate interaction. (c) The 2D and 3D structure diagrams of PRKCA and Glycitein interaction. (d) The 2D and 3D structure diagrams of PRKCA and Mandenol interaction. (e) The 2D and 3D structure diagrams of PRKCA and Rhein interaction. (f) The 2D and 3D structure diagrams of PRKCA and Truflex OBP interaction. (g) The 2D and 3D structure diagrams of PRKCA and Wedelolactone interaction. (h) The 2D and 3D structure diagrams of PRKCB and Atropine interaction. (i) The 2D and 3D structure diagrams of PRKCB and Glycitein interaction. (j) The 2D and 3D structure diagrams of PRKCB and Lucidusculine interaction. (k) The 2D and 3D structure diagrams of PRKCB and Rhein interaction. (l) The 2D and 3D structure diagrams of PRKCB and Wedelolactone interaction.

In order to provide further illustration of how the active compounds bind to the targets, PRKACA, PRKCA, PRKCB, and related active compounds were selected to perform molecular docking as receptors and ligands (Figure 7). PRKACA was a member of the PKA family; meanwhile, PRKCA and PRKCB were members of the PKC family. The three hub genes were enriched in calcium signaling pathway and top ranked in the PPI network. There were hydrogen bonding interactions, *π*-*π* interactions, and hydrophobic were primarily involved between the receptor and ligand. The bind of Obtusin and THR 183, Rhein and ASN 31, GLN 91, Glycitein and LYS 371, GLU 390, ASP 483, Rhein and ASP 484, PHE 485, Wedelolactone and GLU 665, and SER 664 were hydrogen, so their binding affinity were high. The interaction of Ethyl linolenate and active site residues was an alkyl chain, so the docking score was very low, the same situation also occurred in Mandenol and PRKCA.  Table 2 indicated that the binding energies of the ligands to receptors were almost less than -5.0 kcal/mol, which successfully demonstrated that related active compounds could bind well with PRKACA, PRKCA, and PRKCB.

### 3.7. MDs

**Figure 8:**
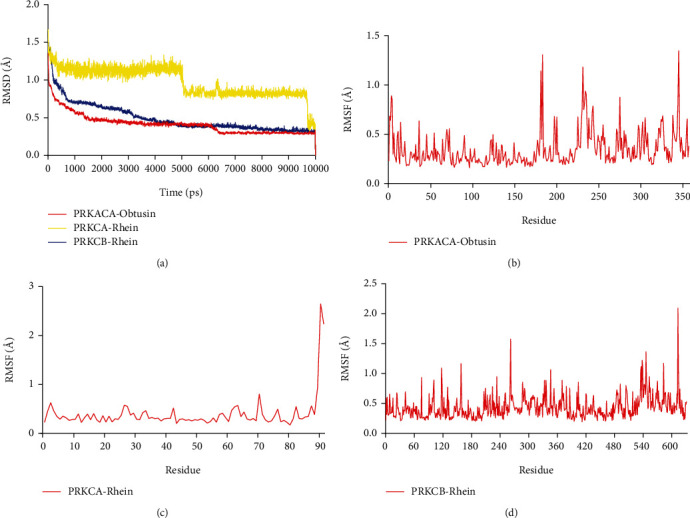
Results of molecular dynamic simulations of the complexes. (a) RMSD of PRKACA-Obtusin, PRKCA-Rhein, PRKCB-Rhein. (b) RMSF of PRKACA-Obtusin. (c) RMSF of PRKCA-Rhein. (d) RMSF of PRKCB-Rhein.

To further investigate the protein-ligand complex, the three complexes with the best molecular docking results were selected for molecular dynamics analysis; the results were showed in Figure 8. Root mean square deviation (RMSD) can evaluate the stability of the trajectory, when the trajectory is steep, it indicates that the system has undergone some kind of violent transformation, and when the trajectory is smooth, it indicates that the system has reached equilibrium. During the initial period, the RMSD of the protein-ligand complexes varied considerably; the RMSD value of PRKACA-Obtusin complex was stable after 1.5 ns; as for the PRKCA-Rhein and PRKCB-Rhein, the stable time was approximately 5 ns, suggesting that at this point, the complex had a stable conformation (Figure 8(a)). Root mean square fluctuation (RMSF) was an indicator to assess the degree of movement of atoms; lower RMSF value meant less drift. Figures 8(b)–8(d) demonstrated that the residues deviation was low. Above all, these results suggested the stability of the complexes.

### 3.8. Experimental Validation

#### 3.8.1. The Inhibitory Effect of CCE on the Proliferation of rCSMCs

**Figure 9:**
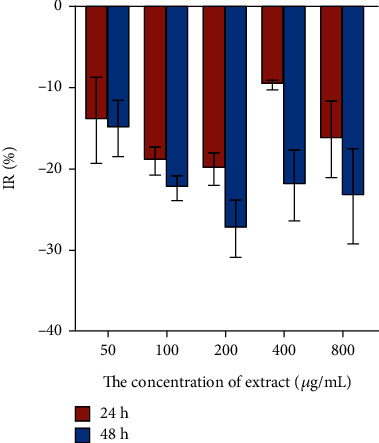
Inhibition rate of CCE on the proliferation of the rat ciliary smooth muscle cells. rCSMCs were treated with CCE at concentrations ranging from 50 *μ*g·mL^−1^ to 800 *μ*g·mL^−1^ for 24 h and 48 h. The data are represented as mean ± SD (*n* = 3).

The inhibitory effect of CCE with different concentrations on the proliferation of rCSMCs at different administration time was shown in Figure 9. When the concentrations of CCE were 50, 100, 200, 400, and 800 *μ*g·mL^−1^, it had no inhibitory effect on the growth of rCSMCs for 24 h and 48 h.

#### 3.8.2. Effect of CCE on NO Production in rCSMCs

**Figure 10:**
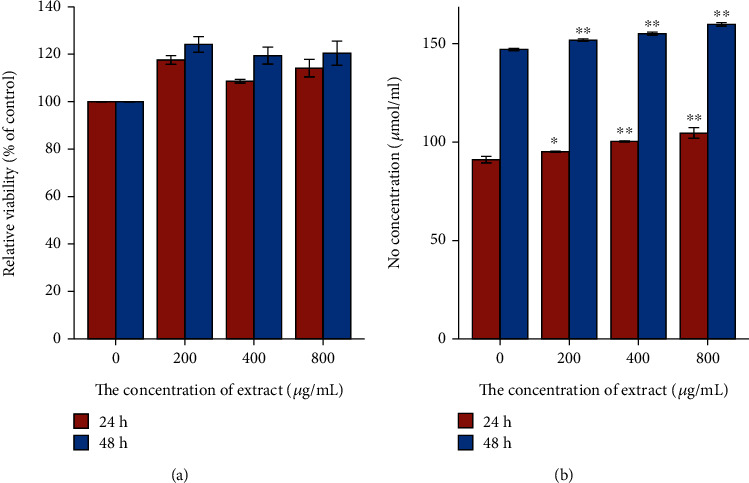
Effect of EEC on (a) relative viability and (b) NO in rat ciliary smooth muscle cells. rCSMCs were treated with 200, 400, and 800 *μ*g·mL^−1^ of CCE for 24 h and 48 h. Cytotoxicity was estimated by the MTT assay, and NO was quantified using the Griess agent. The data are represented as mean ± SD (*n* = 3). ^∗^*p* < 0.05 and ^∗∗^*p* < 0.01.

The concentration of NaNO_2_ solution was taken as the abscissa, and the OD value was taken as the ordinate to perform linear regression. The regression equation was *y* = 0.0045*x* + 0.0603 (*R*^2^ = 0.9956), and the linear range was 0-200 *μ*mol·L^−1^. The effect of CCE on NO production in rCSMCs was shown in Figure 10. CCE was applied to rCSMCs for 24 and 48 hours at doses ranging from 0 *μ*g·mL^−1^ to 800 *μ*g·mL^−1^. There were no appreciable changes in cell viability following CCE at these concentrations (Figure 10(a)). Therefore, rCSMCs were treated with 200, 400, and 800 *μ*g·mL^−1^ of CCE to determine NO production. Compared with the control group, CCE with a concentration of 200 *μ*g·mL^−1^ had a significant increase on the production of NO in rCSMCs (*p* < 0.05), and it had a very evident effect on the content of NO at concentrations of 400 and 800 *μ*g·mL^−1^, when the administration time was 24 h (*p* < 0.01). When the administration time increased to 48 h, CCE obviously elevated the production of NO in rCSMCs with concentrations of 200, 400, and 800 *μ*g·mL^−1^ (*p* < 0.01) (Figure 10(b)).

#### 3.8.3. Effect of CCE on Ca^2+^ in rCSMCs

**Figure 11:**
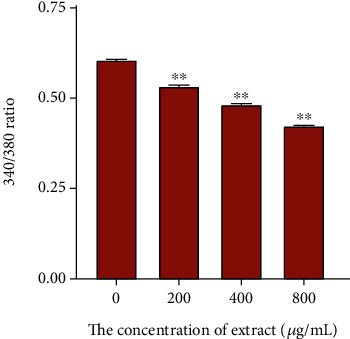
Effect of EEC on Ca^2+^ in rat ciliary smooth muscle cells. Changes of Ca^2+^ were evoked by various concentrations of CCE (200 *μ*g·mL^−1^, 400 *μ*g·mL^−1^, and 800 *μ*g·mL^−1^). The data are represented as mean ± SD (*n* = 3). ^∗^*p* < 0.05 and ^∗∗^*p* < 0.01.

The effect of CCE with different concentrations on Ca^2+^ in rCSMCs was shown in Figure 11. The fluorescence intensity ratio was significantly reduced under the action of CCE at concentrations of 200, 400, and 800 *μ*g·mL^−1^, and within a certain range, the higher the concentration of CCE, the smaller the fluorescence intensity ratio. Compared to the control group, CCE quickly decreased the Ca^2+^ levels in these cells. As a result, Ca^2+^ concentrations were significantly reduced by CCE in a dose-dependent manner.

#### 3.8.4. Changes in Muscle Tension before and after Administration

**Figure 12:**
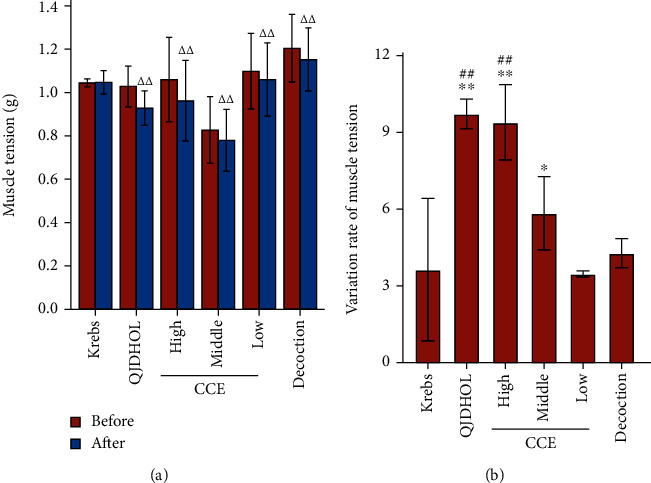
Changes in muscle tension before administration and after administration (a) and the variation rate of muscle tension on the gastric muscle of SD rats (b). The gastric muscle strip specimens performed distinctly different telescopic movements, which were stimulated with Krebs' solution, QJDHOL, decoction, and different concentrations of CCE. The data are represented as mean ± SD (*n* = 6). ^△△^*p* < 0.01 vs. before administration. ^∗^*p* < 0.05 and ^∗∗^*p* < 0.01 vs. Krebs. ^##^*p* < 0.01 vs. decoction.

Figure 12 shows the results of muscle tension and its change rate before and after administration of gastric muscle strips in each group of rats. In this vitro model, SD rats were sacrificed to obtain statistically sufficient data. It can be seen from the results that there was no significant decrease in muscle tension in Krebs' group compared with the muscle tension before administration, while the contraction tension of the other groups was obviously reduced. CCE with medium dose and above had a remarkably enhancement on variation rate of muscle tension in the positive control group (QJDHOL group). There was no significant effect on variation rate of muscle tension in decoction group. Therefore, it showed that a certain concentration of CCE had a direct relaxing effect on the isolated gastric smooth muscle of rats, which could effectively reduce its contraction tension, and its efficacy was better than that of decoction.

#### 3.8.5. Antiasthenopia Effect of CCE In Vivo

**Figure 13:**
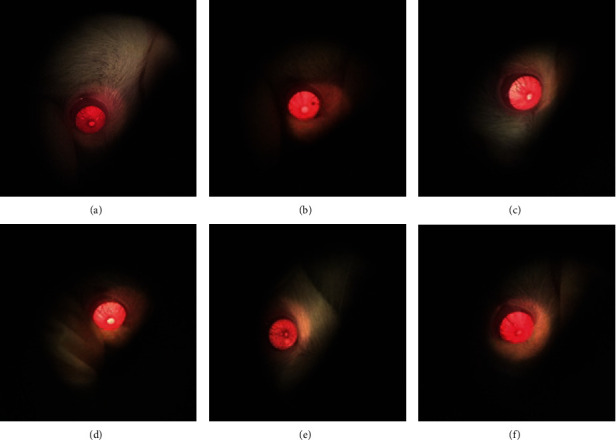
Pupils of rats observed under the ophthalmoscope. (a) Control group. (b) Model group. (c) QJDHOL group. (d) CCE low-dose group. (e) CCE medium-dose group. (f) CCE high-dose group.

**Figure 14:**
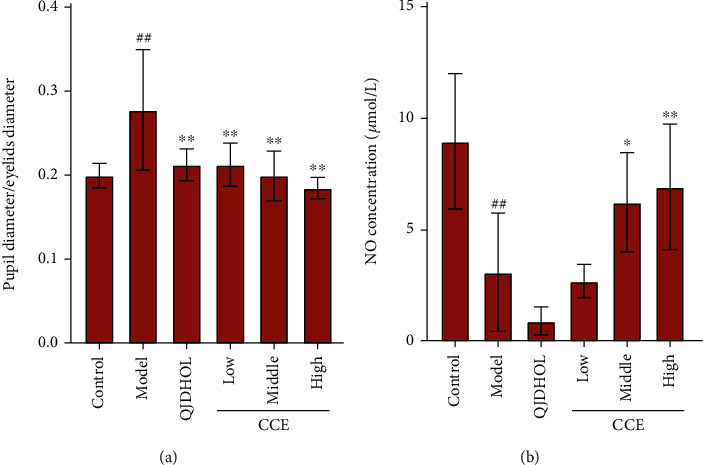
Effect of EEC on (a) the ratio of the pupil diameter to the eyelid diameter in SD rat and (b) NO in the rat ciliary muscle. In this in vivo model, the rats were irradiated with incandescent lamp (100 w) for 15 min. CCE was administered with low dose, medium dose, and high dose for three days before irradiation with incandescent lamp. Intracellular NO levels were measured by the Griess agent. The data are represented as mean ± SD (*n* = 6). ^##^*p* < 0.01 vs. control. ^∗^*p* < 0.05 and ^∗∗^*p* < 0.01 vs. model.

To verify if the effect of CCE could be investigated in vivo, we studied the antiasthenopia effect of CCE (low dose, medium dose, and high dose) on NO levels in irradiation-induced visual fatigue animal experiments. The concentration of NaNO_2_ solution was taken as the abscissa, and the OD value was taken as the ordinate to perform the linear regression. The regression equation was *y* = 0.0064*x* + 0.0771 (*R*^2^ = 0.9940), and the linear range was 0-100 *μ*mol·L^−1^. The pupils of the rats dilated especially in control group compared with the rats that were not irradiated, while the pupils of the rats in other group treated with CCE and QJDHOL retracted a lot (Figure 13). The effect on NO in the supernatant of ciliary muscle homogenate of rats was shown in Figure 14(a). CCE with medium dose and high dose significantly increased intracellular NO levels in rats irradiated with incandescent lamp compared with the model group. There was no significant difference in NO content between the low-dose group and the model group as well as the QJDHOL group. The analysis of the data showed that CCE had not only a contraction on pupil of rats but also a certain effect on increasing the content of NO in the supernatant of rat ciliary muscle homogenate (Figure 14(b)).

#### 3.8.6. qRT-PCR Validation of the Hub Genes

**Figure 15:**
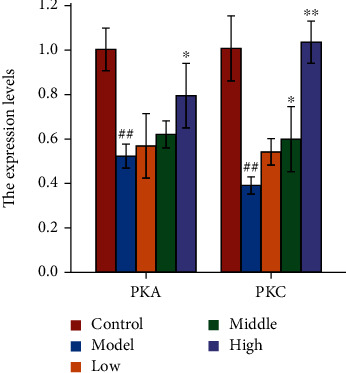
The mRNA expression of PKA and PKC. The data are represented as mean ± SD (*n* = 3). ^∗^*p* < 0.05 and ^∗∗^*p* < 0.01 vs. model. ^##^*p* < 0.01 vs. control.

The expression of hub targets (PKA and PKC) which enriched in calcium signaling pathway was evaluated using qRT-PCR. As shown in Figure 15, compared with the control group, the mRNA expression of PKA was decreased significantly in high-dose group, and PKC were decreased significantly in medium-dose and high-dose group. These results confirmed that after the treatment of CCE, the mRNA expressions of PKA and PKC were inhibited to varying degrees. The primers utilized in our study were listed in Table 3.

## 4. Discussion

Asthenopia is one of the most common eye diseases. The incidence of visual fatigue has been rising annually in recent years, and the patients who suffer from it are also getting younger and younger due to the accelerating social rhythm and increased strain in job, life and other aspects. Asthenopia will not only seriously interfere with the vision and quality of life but also further lead to age-related macular degeneration (AMD), cataract, blindness, and other diseases. Clinical studies have shown that the formation of asthenopia is closely related to eye factors. Excessive use of the eye will lead to ciliary muscle fatigue. With the decline of ciliary muscle function, the ability of the eye to adjust will also decline, resulting in asthenopia. The concentration of calcium ions in smooth muscle is one of the main factors that cause ciliary muscle contraction. Compound chrysanthemum is commonly used clinically to treat asthenopia in China, but the mechanism is not clear. The emergence of network pharmacology provides a new method to study the mechanism of action of drugs and diseases. In this study, we conducted a systemic study using a combination of network pharmacology and experimental verification to explored the potential mechanism and the target of compound chrysanthemum in treating asthenopia.

In this paper, after the intersection of disease genes and drug target genes, 39 active compounds and 593 target genes were found, which play an important role in the treatment of visual fatigue. Through PPI network analysis, we can get the network of genes, and SRC, HSP90AA1, STAT3, DRD2, and ESR1 were identified to be the hub genes. An active compound-target network was constructed; sorted by BC value, Diosgenin, Lucidusculine, 24-Ethylcholest-4-en-3-one, and Rhein may be the active ingredients in CCE treatment of asthenopia. GO analysis identified a wide range of targets involved in peptidyl-tyrosine phosphorylation, membrane raft, and protein tyrosine kinase activity. KEGG analysis showed that the antiasthenopia effect of compound chrysanthemum was related to neuromodulation and cell signal transduction, of which calcium signaling pathway ranked third. Finally, through molecular docking and molecular dynamic, it was verified that PRKACA, PRKCA, and PRKCB can closely bind to related components.

Ciliary smooth muscle relaxation is known to be regulated by cAMP-independent mechanisms such as nitric oxide-mediated relaxation. Therefore, an increase in NO level can cause the smooth muscles in the eye to relax [47]. Nitric oxide is synthesized from L-arginine by NO synthase and is known to relax vascular smooth muscle through the elevation of cGMP that, in turn, mediates a relaxation response [38, 48–51]. NO synthase has been found in ciliary muscle and other ocular tissues [52]. NO plays a significant role in mediating visual accommodation and relaxing the constricted ciliary muscle. Furthermore, prior research has shown that the Ca^2+^ concentration in cultured smooth muscle cells is a primary factor affecting the smooth muscle contraction. In order to maintain a balance between contraction and relaxation in smooth muscle while it is at rest, Ca^2+^ must be strictly controlled within a proper range. Ca^2+^ is a ubiquitous intracellular signal in all eukaryotes, being an important component of cell signaling, and is involved in diversity effects such as neuronal activity, cell motility, etc. [53].

In the present work, the MTT method was used to treat rCSMCs with a range of different concentrations of CCE. The results showed that CCE had no effect on the growth of rCSMCs at these concentrations and could increase the production of NO and reduce the content of Ca^2+^. Moreover, we found that a certain concentration of CCE could relax smooth muscle by the rat gastric smooth muscle in vitro test. Additionally, according to the results of the in vivo antiasthenopia test, it was confirmed that CCE had a constricting effect on the pupil of SD rats and could increase the NO content in homogenate supernatant of rat ciliary muscle. The mRNA expressions of PKA and PKC on the calcium signaling pathway were detected by qRT-PCR; CCE could achieve a therapeutic effect by reducing the mRNA expression of the two proteins.

## 5. Conclusion

With the help of network pharmacology, we found that 39 active compounds and 593 targets were closely related to the effect of compound chrysanthemum in the treatment of asthenopia. The predicted active compounds were validated by performing molecular docking and molecular dynamic simulations with targets. The KEGG analysis indicated that the enriched pathways were related to neuroactive ligand-receptor interaction, the PI3K-Akt signaling pathway, and the calcium signaling pathway. In vitro experiment, our experiment proved that the safety of CCE and preliminarily confirms that CCE could relax ciliary smooth muscles by increasing the concentration of NO and decreasing the concentration of Ca^2+^ to treat asthenopia. In vivo experiment, compound chrysanthemum had not only constricted pupil of rats but also increased the content of NO; meanwhile, the mRNA expressions of PKA and PKC were inhibited to varying degrees. Our research provides a new idea for the treatment of asthenopia.

## Figures and Tables

**Table 1:** The information of the active compounds in CC.

Number	Compound
1	Acacetin
2	Butin
3	Mandenol
4	3′-O-Methylorobol
5	Pratensein
6	Demethylwedelolactone
7	Wedelolactone
8	Luteolin
9	Quercetin
10	Kaempferol
11	Eriodictyol
12	Lucidusculine
13	DFV
14	Baicalein
15	3′-Methoxydaidzein
16	Truflex OBP
17	Diosgenin
18	((1S,5S,7S)-7-Acetoxy-5-isopropenyl-2,8-dimethylene-cyclodecyl) acetate
19	1,3,8,9-Tetrahydroxybenzofurano(3,2-c)chromen-6-one
20	9,10-Dihydroxy-7-methoxy-3-methylene-4H-benzo(g)isochromen-1-one
21	Atropine
22	Glycitein
23	Rhein
24	Toralactone
25	Rubrofusarin
26	Aurantio-obtusin
27	Obtusin
28	Ethyl linolenate
29	Quinizarin
30	4′,5-Dihydroxyflavone
31	Chryseriol
32	Isorhamnetin
33	Artemetin
34	Eupatorin
35	24-Ethylcholest-4-en-3-one
36	Diosmetin
37	Naringenin
38	5,7-Dihydroxy-2-(3-hydroxy-4-methoxyphenyl)chroman-4-one
39	(2R)-7-Hydroxy-2-(4-hydroxyphenyl)chroman-4-one

**Table 2:** Binding energy of active compounds and hub targets.

Target	Compound	Binding energy (kcal/Mol)
PRKACA	Obtusin	-8.8
PRKCA	Ethyl linolenate	-4.7
	Glycitein	-7.2
	Mandenol	-4.6
	Rhein	-8.1
	Truflex OBP	-6.0
	Wedelolactone	-7.3
PRKCB	Atropine	-6.4
	Glycitein	-8.3
	Lucidusculine	-7.4
	Rhein	-9.0
	Wedelolactone	-8.2

**Table 3:** Primers for qRT-PCR.

Gene	Forward (5′-3′)	Reverse (5′-3′)
PKA	GGACAAGCAGAAGGTGGTGAAGC	ACCAGGCACGTACTCCATGACC
PKC	TCCCTGATCCCAAAAGTGAG	AACTTGAACCAGCCATCCAC
GAPDH	CCAGCCCAGCAAGGATACTG	GGTATTCGAGAAGGGAGGGC

## Data Availability

The data used to support the findings of this study are available from the corresponding authors upon request.
